# The Use of NaOH Solutions for Fouling Control in a Membrane Bioreactor: A Feasibility Study

**DOI:** 10.3390/membranes11110887

**Published:** 2021-11-18

**Authors:** Wirginia Tomczak, Ireneusz Grubecki, Marek Gryta

**Affiliations:** 1 Faculty of Chemical Technology and Engineering, Bydgoszcz University of Science and Technology, 3 Seminaryjna Street, 85-326 Bydgoszcz, Poland; igrubeck@pbs.edu.pl; 2 Faculty of Chemical Technology and Engineering, West Pomeranian University of Technology in Szczecin, ul. Pułaskiego 10, 70-322 Szczecin, Poland; marek.gryta@zut.edu.pl

**Keywords:** ceramic membrane, fermentation, fouling, membrane bioreactor design, membrane cleaning, sodium hydroxide, ultrafiltration

## Abstract

Nowadays, the microbial production of 1,3-propanediol (1,3-PD) is recognized as preferable to the chemical synthesis. However, finding a technological approach allowing the production of 1,3-PD in the membrane bioreactor (MBR) is a great challenge. In the present study, a ceramic ultrafiltration (UF) membrane (8 kDa) for treatment of 1,3-PD broths was used. It has been demonstrated that the membrane used provides the stable permeate flux that is necessary to ensure the stability of the fermentation process in MBR technology. It was noticed that the broth pH has a significant impact on both the final 1,3-PD concentration and permeate flux. Moreover, the feasibility of using NaOH for fouling control in the MBR was evaluated. It has been shown that 1% NaOH solution is effective in restoring the initial membrane performance. To the best of our knowledge, this study is the first to shed light onto the possibility of reducing the amount of the alkaline solutions generated during the MBR operation. Indeed, it has been found that 1% NaOH solution can be successfully used several times for both membrane cleaning and to stabilize the broth pH. Finally, based on the results obtained, the technological conceptions of the MBR technology were designed.

## 1. Introduction

Membrane bioreactors (MBRs) technology was installed for the first time in the 1970s for applications such as landfill leachates, ship bilge water and industrial waste [1]. Nowadays, it is the state of the art in biotechnology, receiving extensive academic attention and increasing research for various uses, such as treatment of industrial, municipal, and domestic wastewater, minimization of biological sludge production as well as manufacturing of value-added bioproducts [2,3,4,5,6]. Indeed, it has been reported that the MBRs global market should increase from USD 1.9 billion in 2018 to USD 3.8 billion by 2023 [7]. The well-established applications of MBRs are due to the fact that they offer plentiful benefits, for instance excellent quality of effluent, complete biomass retention, successful bioconversions, improvements of the products yields and productivity as well as low-energy requirements and a low footprint [8,9,10,11,12,13,14].

Roughly speaking, a membrane bioreactor is defined as a system integrating a bioreactor and membrane module in which numerous types of membranes can be applied [12,15]. For instance, ultrafiltration (UF) membranes are an excellent physical barrier to microbes, suspended particles and colloids. Therefore, they allow the fermentation broth (substrate and products) to permeate through the membrane pores, and in consequence, separate it from the biomass, leading to the recycling of cells into the bioreactor. Therefore, UF membranes allow us to eliminate the inhibitory effects of the product and improve the process yield [10]. Conducting a comprehensive literature review (Table A1) indicated that UF membranes are extensively being used in MBRs applied for biological conversions to produce many valuable products, such as lactic acid [15,16,17,18,19,20,21,22], biohydrogen [23,24,25,26,27], biogas [28,29,30], fructose and gluconic acid [31,32,33], propionic acid [34,35,36], ethanol [37,38], succinic acid [39,40], butyric acid [41] and 1,3-propanediol (1,3-PD) [42]. The latter has received increasing attention since it is particularly attractive to industry. Indeed, this value-added material has extended applications in cosmetics, foods, lubricants and polymer (e.g., polyethers and polyurethanes) synthesis [43,44,45,46,47]. As a matter of fact, its microbial production is recognized as more feasible and preferable to the chemical synthesis [48]. Moreover, as it has been pointed out by Celińska et al. [49], much effort is still being devoted to developing several novel directions in 1,3-PD biotechnological production.

From the information available in Table A1, it is evident that the biggest drawback in the current research focused on the MBRs coupled with UF membranes is the limitation mainly to polymeric materials. The reason may be associated with the fact that ceramic membranes have high capital cost [50,51,52,53]. Nonetheless, it should be noted that coupling of ceramic membranes with a bioreactor may enhance the practical application of MBR technology. Undoubtedly, this is due to the fact that ceramic membranes possess several remarkable advantages over conventional polymeric membranes. Indeed, it has been widely documented in the literature [54,55,56,57,58,59,60,61,62] that ceramic membranes offer excellent chemical stability, mechanical strength and high temperature resistance. Therefore, they can be effectively cleaned under harsh environmental conditions without the risk of damaging membranes integrity, thus shortening their service life. Moreover, as has been pointed out by Baruah et al. [63], ceramic membranes are hydrophilic, hence, they are less susceptible to protein adhesion providing higher fouling resistance than commonly employed polymeric membranes made from, e.g., polypropylene. Finally, it should be emphasized that ceramic membranes demonstrate excellent corrosion resistance [64]. Considering the above, it can be inferred that further studies on the use of ceramic UF membranes in membrane bioreactors may allow us to develop scaleup strategies and MBRs implementation in industry.

A major obstacle to the widespread application of MBRs is decrease in the membrane’s permeability during separation processes of biological liquids and suspensions. Indeed, membrane fouling remains the most significant factor that severely limits performance and cost effectiveness of the membrane processes. Overall, fouling is an inevitable and complex phenomenon which progresses through the adsorption of feed components on the membrane surface (cake layer formation) and inside the membrane pores (pore blockage) [65]. As recognized in the literature [17,18], stable flux during filtration is required to ensure the stability of the fermentation process in an MBR. It is worthy of note that the fouling issue in MBRs has been widely discussed in recently published literature reviews [65,66,67,68,69,70,71,72].

Xiao et al. [73] indicated that one of the challenges for full-scale applications of MBRs is more efficient membrane-fouling control. Of all the fouling control strategies, chemical cleaning is the most essential and common way to remove foulants [74,75]. Indeed, it allows us to restore membrane performance and achieve long-term steady operation in MBRs. However, it has to be pointed out that performing efficient chemical cleaning is a prevailing challenge. This is due to the fact that it requires the selection of suitable cleaning agents which remove feed components deposited on the membrane surface and inside its pores [76]. From a practical application point of view, an important issue that must always be considered is the fact that frequent chemical cleaning may lead to changes in the membrane properties, its damage, and consequently, a reduction its lifetime [71]. The importance of this point is that the frequency and duration of cleaning should be minimized by selecting the suitable operational parameters and concentration of chemicals specific to the type of foulant [76]. It should be noted that there is considerably less international literature dedicated to membrane cleaning than to fouling. Indeed, it appears from the literature that, so far, only few papers focused on the cleaning of ceramic UF membranes applied in MBRs have been published (Table A1). Hence, it can be concluded that further investigations on this issue are needed. Nonetheless, this is a serious challenge, since, as has been emphasized by Huang et al. [77], membrane cleaning is requires complex scientific research, the performing of which requires multidisciplinary knowledge.

As a general rule, chemical cleaning agents fall into the following categories: alkalis (e.g., NaOH), acids (e.g., HCl), surfactants, metal chelating agents, enzymes and disinfectants as well as oxidants (e.g., NaOCl) [75,77,78,79,80,81]. It has been frequently reported that alkaline cleaning is effective in removing organic foulants [82,83]. Among alkali cleaning agents, caustic soda (sodium hydroxide, NaOH) is the most aggressive. It allows dissolution of weakly acidic organic matter, and consequently leads to cleavage of polysaccharides and proteins into sugars and amides, respectively [79]. Importantly, summarizing the literature data (Table A1) it can be indicated that NaOH has been used to clean both polymeric [25,41] and ceramic [28,42] UF membranes applied in MBR technology. Our previous investigations [84,85] highlighted the efficiency of NaOH solutions in removing foulants from ceramic UF membranes after filtration of glycerol postfermentation solutions containing 1,3-propanediol as a main product. An important point which should be noted is that the chemical cleaning process generates a waste of spent cleaning solutions which undoubtedly should be considered during the MBRs design stage. On the other hand, several studies [17,22,23,24,25,35,37,38,39,42] have been oriented towards using NaOH solutions to maintain the pH of various fermentation media feeding MBRs at a favorable level (Table A1). This observation suggests the possibility of minimizing or eliminating the generation of waste NaOH by returning it to the bioreactor after membrane cleaning in order to alkalinize the fermenting medium.

Therefore, it can be concluded that research focusing on the use of NaOH solutions for the cleaning of membranes applied in MBRs is significant in its current state. Encouraged by the aforementioned conclusion, in this work we investigated the feasibility of using NaOH solutions for fouling control in a bioreactor coupled with a ceramic UF membrane, applied for 1,3-propanediol production via glycerol fermentation. Moreover, the possibility of using the NaOH waste solutions generated during membrane cleaning to stabilize the pH of fermentation broth was investigated. Hence, the newest insights into the possibility of a significant reduction in the amount of the NaOH solutions generated during the operation of the membrane bioreactor are presented. Finally, the obtained results allowed us to design the technological conception and maintenance of the MBR technology. 

## 2. Materials and Methods

### 2.1. Fermentation

In the present study, *Citrobacter freundii* bacteria were used for the fermentation of glycerol solutions. The bacteria were isolated and characterized by the Department of Biotechnology and Food Microbiology, Poznan University of Life Science, Poland. One liter of the fermentation medium contained (g): glycerol as a carbon source (10.0 or 20.0), yeast extract (2.0), meat extract (1.5), peptone K (2.5), MgSO_4_·7H_2_O (0.58), (NH_4_)_2_SO_4_ (1.2), CaCl_2_·2H_2_O (0.1) and CoCl_2_·6H_2_O (0.013). After sterilization, the liquid medium was inoculated with bacteria in a lag phase (5% vol.). The fermentation process was carried out under anaerobic conditions for 1–3 days in a bioreactor LiFlusGX (Biotron Inc, Bucheon, Korea) with a working volume of 2 L. The medium in the bioreactor was stirred at the agitation speed of 150 ± 5 rpm. The bioreactor was equipped with a control system for automatic regulation of the broth temperature and pH. The broth temperature was equal to 300 K. In order to investigate the impact of the broth pH on the 1,3-PD production, fermentation processes were carried out under medium pH in the range of 7 to 10. The broth pH was controlled by direct addition of NaOH solution (5 M).

### 2.2. Experimental Setup 

**Figure 1:**
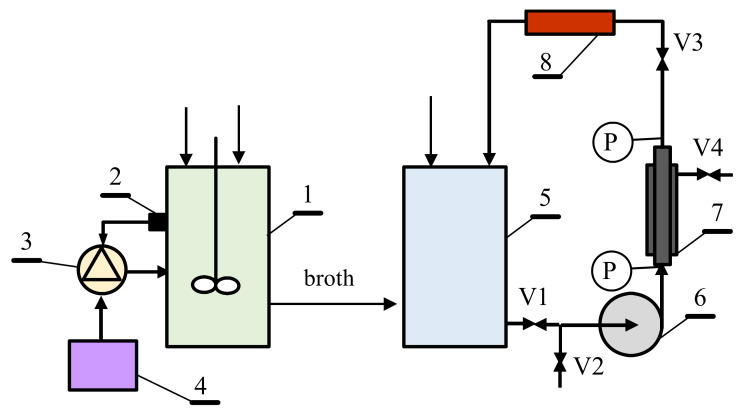
Schematic diagram of a bioreactor coupled with an external installation with the UF membrane module. 1—bioreactor, 2—pH sensor, 3—NaOH dosing unit, 4—NaOH tank, 5—feed tank, 6-pump, 7—membrane module, 8—heater, V1–V4—valves, P—manometer.

The MBR was mainly composed of two complementary units, a bioreactor and external installation with the UF membrane module, sequentially (Figure 1). The tubular, monochannel ceramic membrane (Table 1) used to establish the MBR system was provided by TAMI Industries (Lyon, France). The transmembrane pressure (TMP) was measured using manometers located on the inlet (*P*_1_), outlet (*P*_2_) of the membrane module and manometer on the permeate side (*P_P_*), as follows:

(1) $$TMP=\frac{P_{1}+P_{2}}{2}-P_{P}$$

The permeate flux, *J*, was calculated by measuring the permeate volume collected at a specific time, according to the following equation:

(2) $$J=\frac{dV}{dt}\frac{1}{S}$$

where *V* refers to the permeate cumulative volume in defined timeand *S* refers to the total active membrane area.

The relative flux, *J_r_*, was defined as the ratio between the actual permeate flux and permeate flux *J*_0_ obtained under the same operational conditions:

(3) $$Jr=\frac{J}{J_{0}}\cdot 100\%$$

The feed cross-flow velocity was determined using the quotient of the feed flow rate and inner cross-sectional area of the membrane.

The UF process was performed in cross-flow mode under constant TMP equal to 0.1 MPa. Fermentation broth was pumped through the membrane module with a cross-flow velocity (u) equal to 4.12 m/s which corresponds to the Reynolds number of 26,121. The feed temperature T was maintained at 300 K.

As stated before, our previous research [84,85] has revealed the effectiveness of NaOH solutions in cleaning ceramic UF membranes after treatment of glycerol postfermentation solutions with 1,3-propanediol. In light of this finding, in the present study, after completing each series of the filtration run, the fouled membrane was cleaned according to the procedure presented in Table 2. 

Cleaning efficiency (flux recovery rate, *FRR*) was determined as a ratio of pure water flux after the membrane cleaning *J_c_* to *J*_0_, as follows:

(4) $$FRR=\frac{J_{c}}{J_{0}}\cdot 100\%$$

### 2.3. Analytical Methods

The fermentation broth and permeate were characterized in terms of turbidity, concentration of glycerol, 1,3-propanediol and organic acids as well as number of bacteria. The analytical methods used for this purpose were presented in our previous studies [86,87].

## 3. Results and Discussion

### 3.1. 1,3-Propanediol Formation

**Figure 2:**
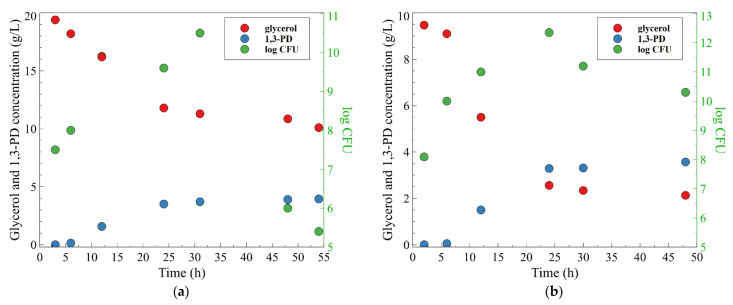
The changes of glycerol and 1,3-propanediol concentrations and number of viable cells of bacteria in the broth during the fermentation process, under conditions of uncontrolled pH. Initial glycerol concentration: (**a**) 20 g/L; (**b**) 10 g/L.

The main product of the glycerol fermentation process with the use of *Citrobacter freundii* bacteria was 1,3-propanediol. The representative time profiles of glycerol and 1,3-PD concentrations as well as the number of viable cells of bacteria (log CFU) present in the bioreactor are shown in Figure 2. It has been determined that the time of bacteria adaptation process was equal to 5–6 h. In the following hours, an increase in log CFU from 7 to 10.5 was noted. After the initial lag phase, the glycerol concentration began to decrease along with gradual increase in the product concentration. The significant changes in the glycerol and 1,3-PD concentrations during the first 31 h of the fermentation process have been noted. Indeed, during this time, an initial concentration of glycerol decreased from 20 to 11.3 g/L, while the concentration of 1,3-PD was equal to 3.71 g/L. In turn, after this time, a significant reduction in the number of bacteria was found (log CFU = 6). Then, the slight changes in the concentration of the substrate and the main product were recorded. Indeed, once the fermentation run was completed, the concentrations of glycerol and 1,3-PD were equal to 10.1 g/L and 3.95 g/L. Comparatively, similar results were obtained for the fermentation process of glycerol with an initial concentration equal to 10 g/L (Figure 2b). Indeed, during 30 h of the process, the glycerol concentration decreased from 10 to 2.34 g/L, while a 1,3-PD concentration of 3.31 g/L was noted. Until the end of the process run, the concentrations of substrate and main product were changed significantly, and after 48 h their values of 2.13 g/L and 3.57 g/L, respectively, were noted. The experimental results reported above allow the indication that the obtained conversation yields were equal to 0.516 mol/mol and 0.549 mol/mol, for initial glycerol concentrations equal to 20 and 10 g/L, respectively. It should be pointed out that the above values of the 1,3-PD yield are in line with those obtained in previous studies [88,89,90], where the glycerol fermentation process to 1,3-PD with *Citrobacter freundii* has been studied. For instance, Barbirato et al. [88] investigated glycerol conversion to 1,3-PD by *Citrobacter freundii* ATCC 8090. The process was conducted at a temperature equal to 30 °C and medium pH of 7. The authors noted a 1,3-PD equal to 0.65 mol/mol and 0.54 mol/mol, for initial glycerol equal to 20 g/L and 70 g/L, respectively.

**Figure 3:**
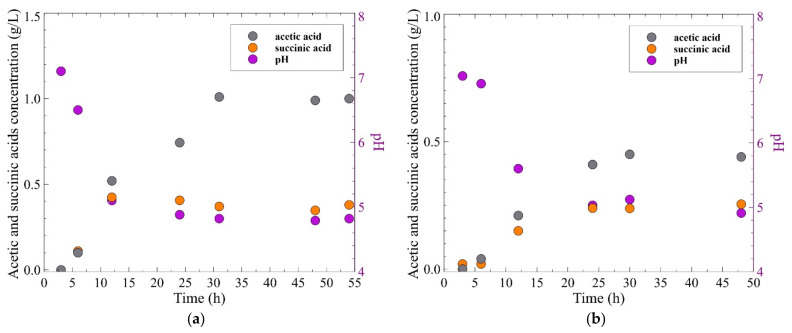
The changes of carboxylic acids concentrations and broth pH during the glycerol fermentation process, under conditions of uncontrolled pH. Initial glycerol concentration: (**a**) 20 g/L; (**b**) 10 g/L.

Incomplete conversion of glycerol was caused by the fact that apart from the main product, carboxylic acids (mainly acetic and succinic acids) were formed. Evidently, formation of these byproducts caused an unfavorable reduction in the broth pH. Figure 3 shows the changes of broth pH and concentration-time profiles of byproducts during the fermentation of glycerol with two initial glycerol concentrations: 20 g/L (Figure 3a) and 10 g/L (Figure 3b). It can be clearly observed that the most significant decrease in the broth pH was noted during the first 24 h of the processes. For an initial glycerol concentration equal to 20 g/L, the medium pH decreased from 7.1 to 4.9, while the concentrations of acetic and succinic acids increased to 0.74 and 0.40 g/L, respectively. Over the next hours, the values of pH and concentrations of above-mentioned byproducts were stabilized. Similar results were obtained for the fermentation of glycerol with an initial concentration equal to 10 g/L. Indeed, it has been found that in the first 24 h, the pH of the medium dropped from 7 to 5 as the acetic and succinic acid concentrations increased to a level of 0.41 and 0.24 g/L, respectively.

**Figure 4:**
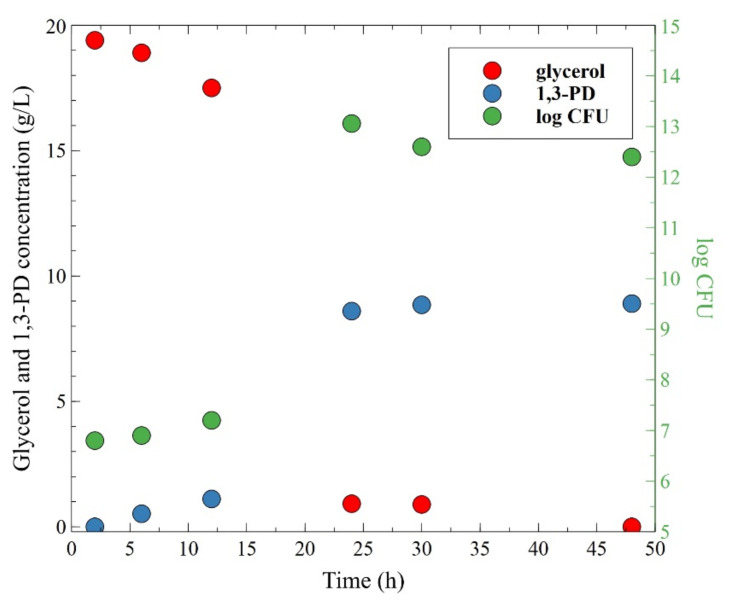
The changes of glycerol and 1,3-propanediol concentrations and number of viable cells bacteria in the broth during the fermentation process with pH regulation (broth pH = 7). Initial glycerol concentration: 20 g/L.

**Figure 5:**
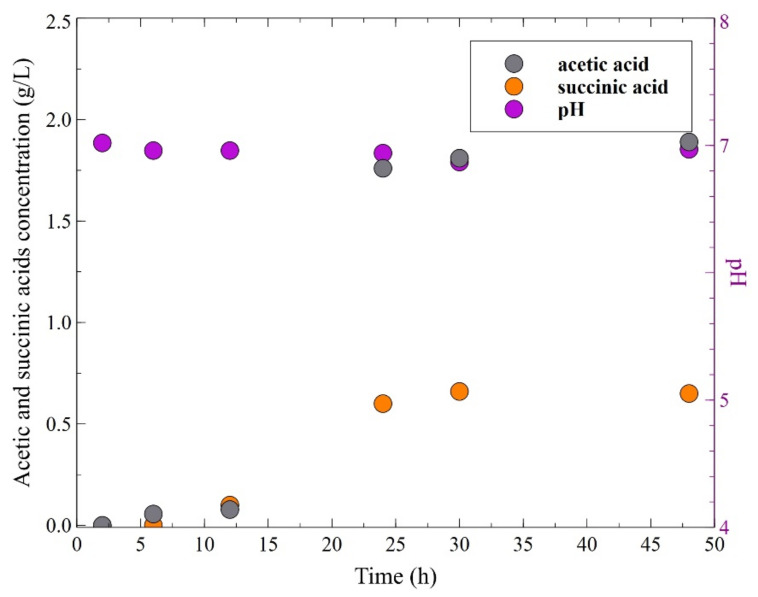
The changes of carboxylic acids concentrations and broth pH during the glycerol fermentation process with pH regulation (broth pH = 7). Initial glycerol concentration: 20 g/L.

In the MBR applications, the concentration of 1,3-propanediol is the first parameter to consider. This is due to the fact that the high final concentration of 1,3-PD is required for both its successful industrial production and effective downstream processing from the fermentation broths [91]. Therefore, in the next stage of the research presented, the effect of the broth pH on the 1,3-PD formation has been investigated. Hence, in order to avoid excessive acidic condition, dosing of NaOH solution to the fermenting medium was applied. The conducted processes showed that the use of the NaOH solution allowed stabilization of the value of medium pH in the range of 6.8–7 (Figure 4), which is considered as the most favorable. Importantly, it has been demonstrated that after 30 h of the process run, more than 95% of the glycerol had been consumed and the 1,3-PD concentration of 8.9 g/L was noted. Moreover, although the metabolites concentration (Figure 5) was higher than that reported in the previously performed fermentation process (Figure 3), the number of bacteria stabilized at the level of 12.4–12.6 log CFU (Figure 4). The results obtained in the present study highlight the importance of regulating the pH of glycerol broth during the production of 1,3-PD through microbial fermentation. Indeed, it has been found that increasing the pH of the medium led to the significant increase in the 1,3-PD concentration (from 3.95 to 8.9 g/L). Worthy of note, this observation corresponds to the previously published reports [88,89,90,92,93], where it was clearly demonstrated that the bioconversion of glycerol to 1,3-PD by *Citrobacter freundii* is strongly affected by the pH of culture medium. For instance, in [89] it was found that the maximum concentration of 1,3-PD obtained via glycerol fermentation performed under pH control was over five-fold higher than that reported during the process without pH regulation. In turn, Metsoviti et al. [90] demonstrated that applying the suitable control of medium pH leads to an almost two-fold increase in the final concentration of 1,3-PD. 

In order to investigate the effect of the broth pH on the number of bacteria cells in the bioreactor, the broth obtained after 24 h of glycerol fermentation (20 g/L, pH = 7) was placed in 250 mL flasks. Subsequently, suitable amounts of NaOH solution were added to the flasks to obtain defined pH values. Then, the flasks were placed in an incubator (T = 300 K) and samples were taken for inoculation every 30 min. The obtained results are presented in Table 3. It has been found that the number of viable cells of bacteria in broths with pH equal to 7 and 8.5 did not change significantly during the test performed. Therefore, from the results presented so far, the conclusion can be drawn that it is advisable to use NaOH solutions in an amount that increases the broth pH up to 8.5. Above this value, slowing down of bacterial cells’ growth begins.

### 3.2. Ultrafiltration Performance

The results of the fermentations carried out have demonstrated very good reproducibility. Indeed, for a medium with an initial glycerol concentration of 20 g/L and pH maintained at value equal to 7, after 24 h of the process the noted concentrations of glycerol and 1,3-propanediol were in the range of 0.26–0.41 g/L and 8.5–8.7 g/L, respectively. Once the fermentation process run was complete, the obtained broth was transferred from the bioreactor to the UF installation tank. 

As expected, it has been noted that the membrane used was efficient in the clarifying of the fermentation broths. Indeed, a sterile permeate with a turbidity equal to 0.1 NTU was obtained. It should be pointed out that the compositions of the feed and permeate were very similar. This is due to the fact that the used UF membrane (8 kDa) separated mainly the high molecular compounds. A detailed investigation focused on the efficiency of the membrane used and the impact of the process parameters (transmembrane pressure and feed flow rate) on the permeate flux during the filtration of 1,3-PD broths was presented in our previous work [85]. 

**Figure 6:**
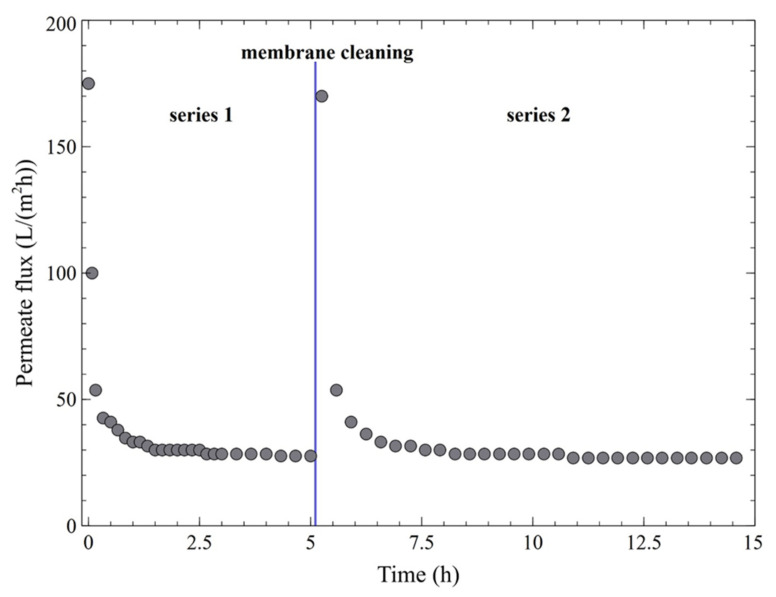
Changes in the permeate flux during the UF process of a fermentation broth (pH = 7). The blue line represents cleaning of the membrane module with 1% NaOH solution (15 min).

In the present research, it has been determined that using the above-mentioned process parameters (TMP = 0.1 MPa, u = 4.12 m/s and T = 300 K), the pure water flux *J*_0_ for a clean membrane was equal to 178 L/(m^2^h). However, during the UF process of 1,3-PD fermentation broth, the membrane performance decreased significantly due to the fouling phenomenon. As expected, the most notable permeate flux decline was noted for the initial series phase. Indeed, a permeate flux of 42 L/(m^2^h) after 20 min of the experiment run was noted. Finally, after about 180 min, the steady-state permeate flux was equal to 27 L/(m^2^h) (Figure 6, series 1) which constitutes 15% of its maximum value. It should be pointed out that the results obtained in the present study are consistent with previous findings in the literature [17,18,20,21,22,28,36,42], wherein a significant reduction in the UF membrane’s performance applied in MBRs has been reported. For instance, Fan et al. [17] have investigated continuous lactic acid fermentation in an MBR with a ceramic UF membrane (a nominal cutoff equal to 100 kDa) (Table A1). The authors have demonstrated that during the fermentation process, the permeate flux strongly decreased from 180 to 30 L/(m^2^h). In a subsequent paper [18], it was found that the flux of a ceramic hollow-fiber membrane with a nominal pore size of 40 nm used in an MBR for lactic acid production (Table A1) dropped from 140 to 72 L/(m^2^h) during the first hour of the process run and declined to 60 L/(m^2^h) during the subsequent 5 h.

**Figure 7:**
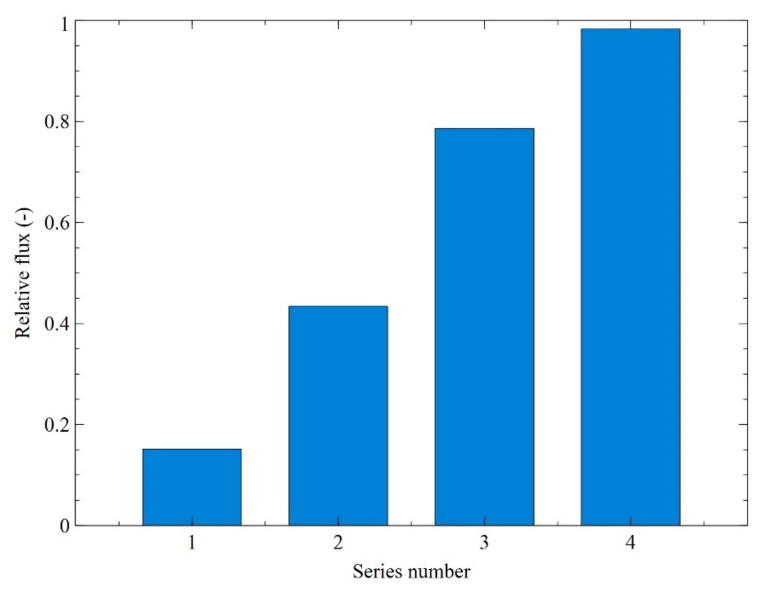
Changes in the relative flux of UF membrane. Relative flux: 1—steady-state, after 180 min of the broth filtration process, 2—after membrane rinsing with water, 3—after membrane cleaning with 1% NaOH solution (5 min), 4—after membrane cleaning with 1% NaOH solution (10 min).

Once the first filtration run was completed, membrane cleaning was performed (Figure 6, the blue line). For this purpose, in the first procedure stage the broth was replaced with distilled water in the feed tank. As shown in Figure 7, this led to an increase in the relative flux to about 43%. In the next step, the installation was rinsed with 1% NaOH solution for 5 min. As a result, a relative flux equal to about 78% was reported. Finally, it has been demonstrated that cleaning the installation with 1% NaOH solution for another 10 min allowed us to recover the initial membrane performance (*FRR* = 100%).

On the basis of the results presented and other investigations mentioned above, it is apparent that NaOH is an effective cleaning agent in restoring the initial performance of the ceramic UF membranes after the filtration processes of fermentation broths. However, it has been found that the effect of the NaOH solution is short-lived and performing membrane cleaning requires cyclical repetition (Figure 6, series 2). Therefore, it can be inferred that for a bioreactor with continuous product discharge, the performance of the UF membrane decreases rapidly once the broth makes contact with the membrane module. Indeed, after 20 min of the second series run, the flux decreased from 173 L/(m^2^h) to 41 L/(m^2^h), and finally, a steady state permeate flux of 25 L/(m^2^h) was noted. Analyzing the data shown in Figure 6, it can be concluded that the ceramic UF membrane used in the presented investigation provides the stable permeate flux that is necessary to ensure the stability of the fermentation process in a MBR [17,18].

**Figure 8:**
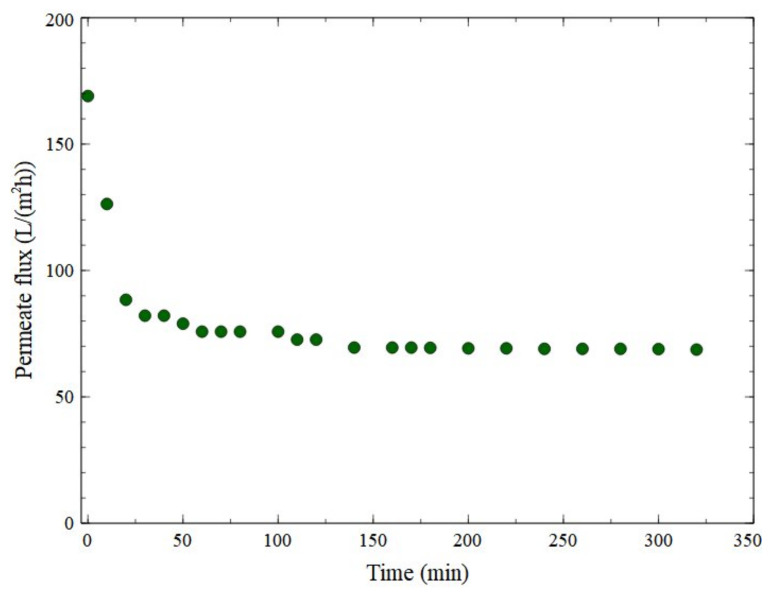
Changes in the permeate flux during the UF process of a fermentation broth (pH = 8.5).

Although it is well-known that the feed pH may significantly affect the flux behavior, to the best of our knowledge, studies devoted to the influence of the fermenting medium pH on the ceramic UF membranes performance are very limited. In our previous study [84] we demonstrated that increasing the broth pH may lead to changes in the interactions between the feed and membrane, resulting in a significant increase in the permeate flux of the ceramic UF fine membrane (450 Da) during the filtration of 1,3-PD fermentation broths. Indeed, in the above-mentioned study we found that the most favorable feed pH allowing to obtain the highest filtration process performance is equal to 9.4. However, it was observed during present experimental investigation (Table 3) that increasing the pH of broth to the values equal to 9.4 and 10 led to a significant reduction in the number of bacterial cells in the fermenting medium. This result clearly indicates that increasing the broth pH above the value of 8.5 is unfavorable for efficient MBR operation. In light of these data, in order to investigate the impact of the broth pH on the steady-state permeate flux and fouling intensity, the UF process of broth with pH equal to 8.5 was carried out (Figure 8). It was confirmed that the rate of fouling is controlled by the medium pH. Indeed, it has been noted that increasing the broth pH from 7 to 8.5 led to an increase in the steady-state permeate flux from 25 to 68 L/(m^2^h). This can be explained by the fact that, as a result of the metal oxide making contact with the feed, the membrane surface groups may undergo the dissociation process, which, in turn, leads to changes in the membrane surface charge and mechanisms of solute adsorption [94,95]. Consequently, it can be assumed that increasing the broth pH led to reduction in the intensity of cake deposition on the membrane surface, resulting in a significant increase in the permeate flux. Our results have some similarities with findings presented in the literature [84,95,96], where the significant impact of various feed pH levels on the ceramic UF membranes permeability was demonstrated. For instance, Lobo et al. [95] used tubular ceramic membranes with cutoffs equal to 50 and 300 kDa for ultrafiltration of a model oil-in-water emulsion. The authors found that at low feed pH values, the permeate flux decreased drastically, since membranes become positively charged leading to adsorption of anionic surfactants onto the membrane surface and flux decline. 

**Figure 9:**
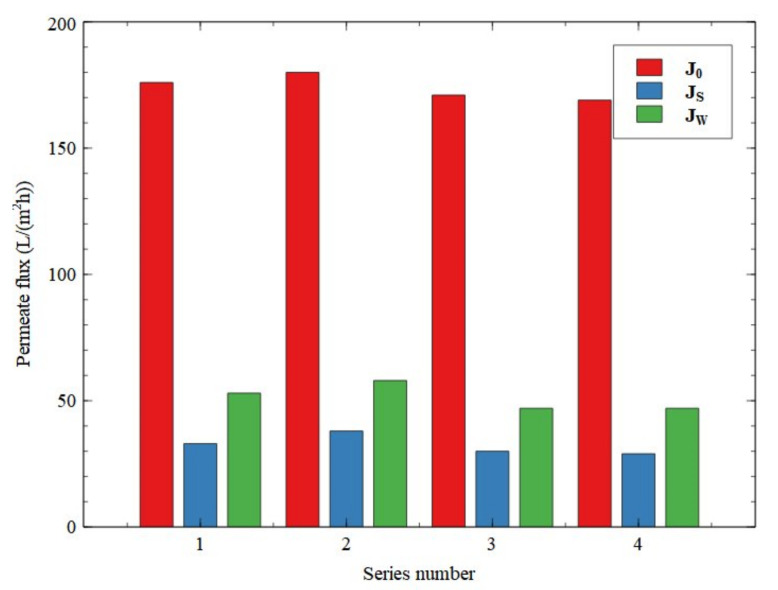
Changes in the performance of the UF membrane during the cyclically repeated operation: filtration-rinsing with 1% NaOH solution. *J*_0_—initial (maximum) permeate flux, *J*_s_—steady-state permeate flux, *J*_w_—permeate flux after membrane rinsing with water.

In order to investigate the performance stability of the membrane used, the filtration-cleaning cycle with 1% NaOH solution was repeated several times (Figure 9). Conducting the presented experiments indicated that the obtained values of the steady-state permeate flux are repeatable. Indeed, after each of the membrane cleaning procedures were performed, the recovered flux was at the same level (170 L/(m^2^h)) as that noted at the beginning of the study (Figure 7). An important result found in the current investigation is that the procedure of membrane cleaning being repeated several times did not have an adverse effect on the membrane performance and separation properties.

We used 1% NaOH solution (1 L) to rinse the UF installation. Once the module cleaning was completed, the solution was poured into the tank and reused in the next cycle (Figure 9). It must be recognized that although the NaOH solution was used several times, it retained its properties and the membrane initial performance was restored during each cleaning run. This noteworthy result indicates that it is possible to reduce the waste of cleaning solutions by their multiple use. Moreover, in the last two series shown in Figure 9, the broth pH was stabilized by dosing NaOH solution that had previously been used to clean the installation. Importantly, this did not affect 1,3-PD formation, and likewise in the case of using pure NaOH solution, 1,3-PD with a concentration of 8.4–8.5 g/L was obtained after 24 h of the fermentation process. 

### 3.3. Design and Maintenance of MBR Facility: Technological Conception

**Figure 10:**
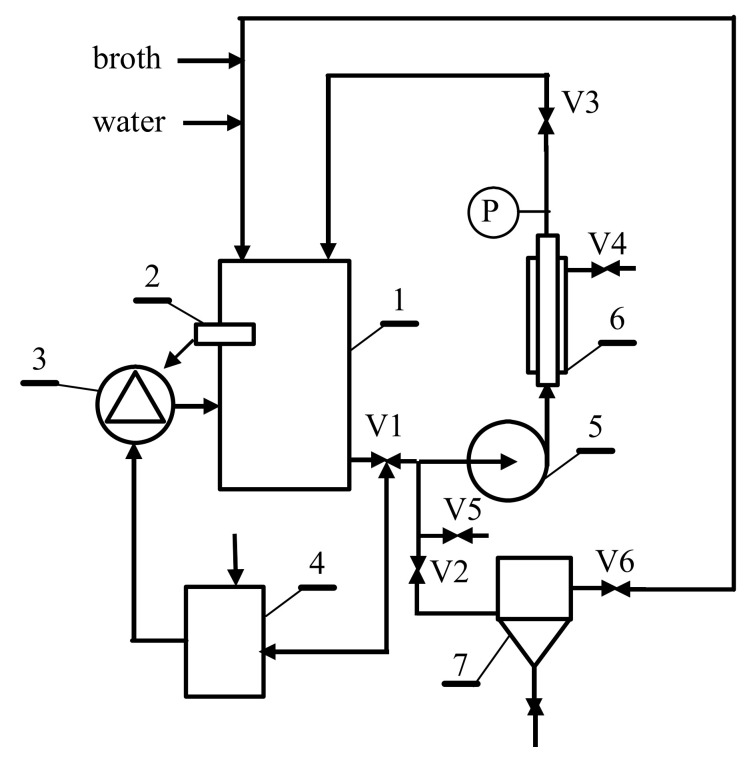
The technological conception of UF installation with a membrane-cleaning system with NaOH solution. 1—feed tank, 2—pH sensor, 3—NaOH dosing unit, 4—NaOH tank, 5—pump, 6—membrane module, 7—retentate tank, V1–V6—valves, P—manometer.

**Figure 11:**
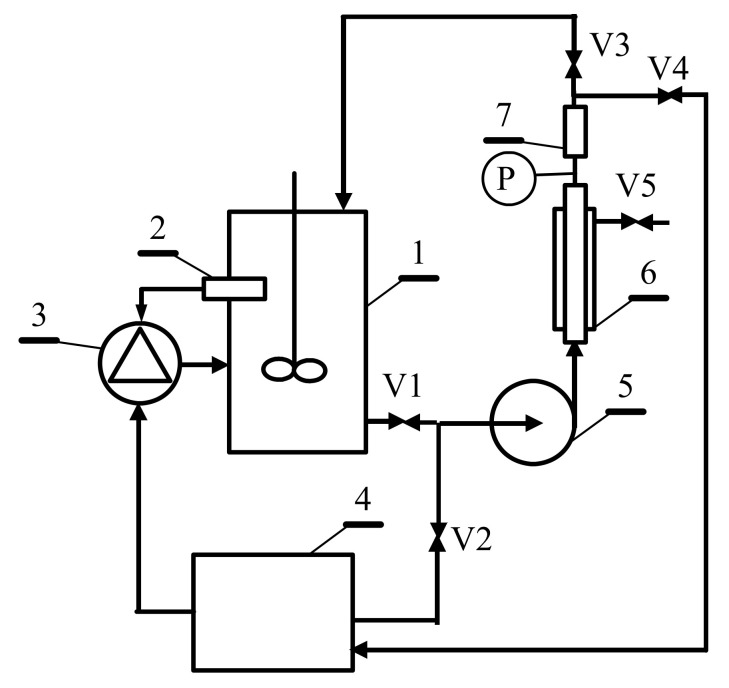
The technological conception of membrane bioreactor with cleaning system with NaOH solution. 1—bioreactor, 2—pH sensor, 3—NaOH dosing unit, 4—NaOH tank, 5—pump, 6—membrane module, 7—pH sensor, V1–V5—valves, P—manometer.

Finding a technological approach allowing the production of 1,3-propanediol via glycerol fermentation in MBRs is a great industrial challenge. The amount of NaOH solution used in the operation of the membrane bioreactor can be significantly reduced. For this purpose, several variants of appropriately designed MBR constructions have to be used. Two technological conceptions of MBR design are schematically shown in Figure 10 and Figure 11. 

The technological solution presented in Figure 10 can be used for both batch and fed-batch fermentation processes. According to the design, the broth from the bioreactor is transferred to the feed tank (1) and is filtered until the minimum volume of the feed in the tank is achieved. The obtained retentate is removed or discharged to a decanter (7), from which, subsequently, it is recycled in the next filtration cycle, leading to an increase in the degree of filtered broth recovery. After the filtration process, the module is prerinsed with water and then with NaOH solution, which, after cleaning, is returned to the tank (4). By implementing this solution, the pH of the feed can be increased. According to the results obtained in the present study (Figure 8), this will lead to a reduction in membrane fouling, and thus, allows us to obtain a higher performance of the UF process.

The second technological solution (Figure 11) refers to the UF installation directly combined to the bioreactor. Similar to the system shown above, it can be used for both continuous and fed-fermentation processes. In the first-mentioned method, the broth flows continuously through the membrane module, and a volume of fresh substrate equal to that of the obtained permeate is fed to the bioreactor. The implementation of the demonstrated solution requires the selection of the UF membrane surface and process parameters ensuring the dilution rate resulting in bioreactor operating conditions similar to those in the biostat. The results obtained in the present work (Figure 9) showed that cleaning of the ceramic UF membrane can be performed every few days without damaging the membrane. For this purpose, after closing valve V1 and opening V2, the NaOH solution displaces the fermentation broth in the system. When the pH (sensor 7) exceeds the value of 8, valve V3 is closed and valve V4 opens, which allows recirculation of the NaOH solution to the tank. Once cleaning is completed, valve V2 is closed and valve V1 is opened, which allows the displacement of the NaOH solution by the broth. When the pH value falls below 8.5 (sensor 7), V4 closes and V3 is opened, allowing the UF process to resume. A slight excess of base will flow into the bioreactor, so it is advantageous to stop the pH stabilization during the operation of the membrane cleaning. This will lead to a decrease in the pH below the value of 7 for a short period of time, and more NaOH solution will be added to the broth, without exceeding the pH of 8. In turn, in the case of the fed-fermentation process, the installation with the UF unit is turned on periodically when the concentration of the dosed substrate (e.g., glycerol) in the broth is close to zero. Starting the UF process will reduce the broth volume, which is supplemented by adding another dose of substrates, and the UF installation is switched to the cleaning cycle.

## 4. Conclusions

Finding a technological approach allowing the production of 1,3-propanediol in a membrane bioreactor is a great challenge. Indeed, a major issue hindering industrial production of 1,3-PD using MBR technology is the membrane-fouling phenomenon. Based on our experiments, it can be established that the integration of the bioreactor with a ceramic UF membrane for production of 1,3-PD via glycerol fermentation can be successfully applied. Indeed, it has been demonstrated that the ceramic membrane with a cutoff equal to 8 kDa provides stable permeate flux that is necessary to ensure the stability of the fermentation process in the MBR. Moreover, the presented study provides a strategy for the minimizing of intensive fouling. Indeed, the results obtained clearly show that increasing the medium pH from 7 to 8.5 led to increasing of the steady-state permeate flux from 25 to 68 L/(m^2^h). Moreover, the feasibility of using NaOH solution for fouling control in a membrane bioreactor was demonstrated. It has been shown that 1% NaOH solution is an effective cleaning agent in restoring the initial performance of membrane. To the best of our knowledge, this study is the first to shed light onto the possibility of a significant reduction in the amount of the NaOH solutions generated during the operation of the membrane bioreactor. Indeed, it has been found that 1% NaOH can be successfully used several times for both cleaning the membrane and stabilizing the pH of fermentation broths. These results are particularly important for the economic aspects of MBR technology. Finally, this study was the first to demonstrate the technological conceptions of 1,3-PD production in an MBR coupled with an external ceramic UF membrane. Therefore, all the presented results increase the opportunity of practical implementations of 1,3-PD production in membrane bioreactors via glycerol fermentation. Finally, we would like to emphasize that extensive research is certainly needed to investigate and explore an economic assessment of the presented technological conceptions.

## Figures and Tables

**Table 1:** Technical data on the UF membrane used in this study.

Parameter	Unit	Value
Number of channels	(-)	1
Cutoff	(kDa)	8
External diameter	(mm)	10
Channel diameter	(mm)	6
Length	(mm)	220
Area	(m^2^)	3.8 × 10^–3^
Support material	(-)	TiO_2_
Membrane material	(-)	ZrO_2_

**Table 2:** Operating conditions for membrane cleaning.

Step	Q (dm^3^/h)	TMP (MPa)	T (K)	t (min)
Distilled water rinsing	350	0	303	5
1% NaOH cleaning				5
Distilled water rinsing				5
1% NaOH cleaning				10
Distilled water rinsing				5

**Table 3:** Impact of broth pH on the number of bacteria in a fermenting glycerol solution.

Broth pH	Log CFU/mL		
	Start	t = 30 min	t = 60 min
7.0	12.62	12.64	12.65
8.5	12.61	12.64	11.83
9.4	12.63	11.72	10.55
10.0	12.60	0	0

## Data Availability

The data presented in this study are available on request from the corresponding author. The data are not publicly available due to the institutional repository being under construction.

## Appendix A

**Table A1:** Application of UF membranes in MBR technology: literature review.

Bioconversion Process				Membrane Characteristic						Membrane Cleaning Agents	Ref.
Main Product	Carbon Source	Microorganism	Buffer	Module Configuration	Membrane Type	Material	Pore Diameter (µm) or Cut-Off (kDa)	Length (m)	Surface Area (m^2^)		
lactic acid	glucose	*Lacticaseibacillus paracasei*	NH_4_OH	external	hollow fiber	PS ^1^	300 kDa	n/a	0.2500	n/a	[15]
lactic acid	glucose	*Lactobacillus rhamnosus*	Ca(OH)_2_	external	NI	organic	1 kDa	n/a	16.0000	n/a	[16]
lactic acid	glucose	*Bacillus coagulans*	NaOH	external	hollow fiber	ceramic	0.04 µm	n/a	0.0330	n/a	[17]
lactic acid	glucose	*Bacillus coagulans*	n/a	external	hollow fiber	ceramic	0.04 µm	0.190	0.0330	alkaline solution (1% Asiral, Asiral Industrie-Reiniger GmbH, Neustadt, Germany)	[18]
lactic acid	lactose, glucose and galactose	*Lactobacillus bulgaricus*	NH_4_OH	external	hollow fiber	PS ^1^	30 kDa	0.268	0.0350	n/a	[19]
lactic acid	glucose	*Bacillus coagulans*	n/a	external	tubular	ceramic	0.05 µm, 100 kDa and 20 kDa	n/a	0.0040	n/a	[20]
lactic acid	lactose	*Lactococcus lactis*	NH_4_OH	submerged	hollow fiber	PVDF ^2^	0.04 µm	n/a	0.0200	-	[21]
lactic acid	glucose, fructose and sucrose	*Lactobacillus rhamnosus*	NaOH	external	hollow fiber	PES ^3^	20 kDa	n/a	0.1800	250 ppm NaOCl (backwashing)	[22]
biohydrogen	glucose	*Clostridium beijerinckii*, *Clostridium pasteurianum* and Enterobacter sp.	NaOH	submerged	hollow fiber	PTFE ^4^	0.10 µm	n/a	0.1950	n/a	[23]
biohydrogen	glucose	*Clostridium*, *Enterobacter* and *Ethanoligenens*	NaOH	submerged	hollow fiber	PTFE ^4^	n/a	n/a	n/a	n/a	[24]
biohydrogen	glucose	n/a	NaOH	submerged	submerged tubular	PVDF ^2^	250 kDa	n/a	0.0400	NaOH (pH = 12) and HNO_3_ (pH = 2)	[25]
biohydrogen	n/a	*Clostridium thermopalmarium* and *Clostridium butyricum*	HCl and NaOH	submerged	fiber	n/a	0.05 µm	0.490	n/a	n/a	[26]
biohydrogen	glucose	n/a	n/a	submerged	hollow fiber	PVDF ^2^	0.04 µm	0.215	0.1000	not performed	[27]
biogas	n/a	n/a	n/a	external	tubular	ceramic	150 kDa	0.300	0.1000	2% NaOH and 1% HNO_3_	[28]
biogas	n/a	n/a	n/a	submerged	hollow fiber	n/a	0.03 µm	0.250	0.5000	14% *w*/*v* (200 ppm) NaOCl, 1 g/L NaOCl and 1 g/L C_6_H_8_O_7_	[29]
biogas	n/a	n/a	NaHCO_3_	submerged	hollow fiber	n/a	0.04 µm	n/a	0.0470	2 g/L citric acid and NaOCl solution containing 2 g/L effective chlorine	[30]
fructose and gluconic acid	sucrose	*Saccharomyces cerevisiae*	acetate buffer	n/a	n/a	n/a	100 kDa	n/a	n/a	n/a	[31]
fructose and gluconic acid	sucrose and glucose	*Aspergillus niger*	acid/acetate buffer	submerged	n/a	regenerated cellulose	100 kDa	n/a	n/a	n/a	[32]
fructose and gluconic acid	sucrose and glucose	*Aspergillus niger*	acetate buffer	submerged	n/a	regenerated cellulose	100 kDa	n/a	n/a	n/a	[33]
propionic acid	glycerol	*Propionibacterium thoenii*	NaOH	external	n/a	mineral	500 kDa	n/a	0.0150	n/a	[34]
propionic acid	lactose	*Propionibacterium* *acidici-propionici*, *Propionibacterium thoenii*, *Propionibacterium jensenii*, *Propionibacterium freudenreichii*, *Lactococcus lactis*	NaOH	external	n/a	n/a	300 kDa	n/a	1.670 and 34.200	n/a	[35]
propionic acid	glucose	*Propionibacterium acidi-propionici*	NH_4_OH	n/a	tubular	ceramic	NI	0.640	0.1000	n/a	[36]
ethanol	sucrose	*Saccharomyces cerevisiae*	NaOH	external	tubular	PES ^3^	0.02 µm	n/a	0.1000	500 mg/L H_2_O_2_ and NaOCl solution containing 100 mg/L active chlorine	[37]
ethanol	saccharose	*Saccharomyces cerevisiae*	NaOH	external	tubular	PPP ^5^	25 kDa	n/a	0.0250	n/a	[38]
succinic acid	glucose	*Actinobacillus succinogenes*	NaOH	external	n/a	ceramic	300 kDa	n/a	0.1600	n/a	[39]
succinic acid	glucose	*Actinobacillus succinogenes* and *Mannheimia succiniciproducens*	n/a	external	hollow-fiber	n/a	300 K	n/a	0.1000	n/a	[40]
butyric acid	glucose	*Clostridium tyrobutyricum*	n/a	external	n/a	PS ^1^	100 kDa	n/a	n/a	10 g/L NaOH and 10 g/L NaOCl	[41]
1,3-PDO	glycerol	*Citrobacter freundii*	NaOH	external	tubular	ceramic	8 kDa	n/a	0.0038	1% NaOH	[42]

polysulfone; ^1^ polyvinylidene fluoride; ^2^ polyethersulfone; ^3^ polytetrafluoroethylene; ^4^ poly(phenylenephthalimide) ^5^.
